# From secondary special needs education to the labor market: latent trajectories and inequalities in employment participation

**DOI:** 10.5271/sjweh.4292

**Published:** 2026-07-01

**Authors:** Robert Ciliacus, Fabio Porru, Alex Burdorf, Merel Schuring

**Affiliations:** 1 Department of Public Health, Erasmus University Medical Center, The Netherlands.

**Keywords:** gender disparity, group-based trajectory modeling, labor market integration, migration-related disparity, NEET, school-to-work transition, special educational need

## Abstract

**Objectives:**

Individuals with disabilities continue to face barriers to labor market inclusion. The transition from secondary special needs education plays a critical role in shaping long-term employment outcomes. Early labor market disparities are particularly concerning given their lasting impact on health and well-being. This study aims to: (i) assess employment participation after leaving special needs education, (ii) identify distinct employment trajectories, and (iii) examine how gender and migration background are associated with these trajectories.

**Methods:**

This longitudinal study used national registry data from Statistics Netherlands to examine employment outcomes among individuals transitioning from secondary education between 2016 and 2023, with focus on those in special needs education. Employment status was available monthly for up to seven years post-transition. Group-based trajectory modeling was applied separately for each educational track within secondary special needs education to identify patterns of labor market participation over time. Gender and migration background were subsequently examined as predictors of trajectory group membership using multinomial logistic regression models.

**Results:**

Employment participation after seven years was substantially lower among former special needs education students (10–53% across tracks) than among those from regular ‘practice-based’ education (70%). After special needs education, employment participation was initially highest among individuals transitioning from the ‘labor market integration’ track, but plateaued around 45%. In contrast, graduates from the ‘post-secondary education’ track, primarily aimed at continued studies, showed steady improvement, leading to better long-term employment outcomes (53%). Within each educational track, 3–5 distinct latent employment trajectories were identified. Post-hoc analyses indicated that women and individuals with a migration background were consistently more likely to follow less favorable patterns.

**Conclusion:**

Educational pathways alone do fully not account for labor market disparities. Outcomes are shaped by the intersection of education and identity characteristics. These findings highlight the need for individualized vocational support and deliberate, equity-focused guidance during educational transitions.

To date, 188 countries, including The Netherlands, have ratified or acceded to the UN Convention on the Rights of Persons with Disabilities (1). This convention promotes equal rights and full inclusion of people with disabilities, including the right to earn a living through employment of their own choosing in a labor market that is open, inclusive and accessible (2). Equitable access to schooling, vocational education and employment opportunities for students with special education needs is a cornerstone of these international commitments. Beyond equity and human rights commitments, governments increasingly promote higher lifelong labor market inclusion as an economic imperative, particularly in the context of labor shortages, an ageing population, and the sustainability of welfare systems. Despite ongoing efforts, large disparities in employment participation between people with and without disabilities persist, both nationally and internationally (3, 4). Consequently, people with disabilities often remain sidelined in the labor market, even though many are both capable and willing to work. These labor market inequalities emerge early, as graduates from special needs education are more likely to experience poorer employment outcomes after leaving school compared to their peers in mainstream education (5, 6). As more students are identified as having special educational needs (7), a growing share of young people may face systemic barriers to labor market inclusion. Gaining deeper insight into the employment participation of individuals after following distinct special needs education tracks may help clarify how educational pathways relate to labor market access and inform strategies to promote inclusive employment policies.

These early labor market disparities are particularly concerning given the strong relationship between employment and health. Participation in paid employment is generally associated with better health outcomes (8, 9). Conversely, unemployment has consistently been linked to poorer, particularly mental, health (10, 11), and may disproportionately impact individuals with pre-existing health issues (12). At the same time, ill health itself reduces the likelihood of entering and sustaining paid employment (13). The interplay between employment and health may thus create a self-reinforcing cycle, particularly for individuals with disabilities: unemployment exacerbates health problems, while declining health further reduces the chances of securing paid employment, intensifying the cycle of exclusion.

A successful school-to-work transition is essential in order to gain access to employment opportunities and foster long-term career development and sustainability (14–16). Beyond economic benefits, early labor market integration supports key developmental processes in emerging adults, including coping skills, self-efficacy, and identity formation (17–20). Conversely, a poor start in the labor market can have long-lasting scarring effects, hindering future career progression, diminishing quality of new employment opportunities, and increasing the risk of unemployment later in life (21–23). Effectively supporting the transition from a special needs education to the labor market is therefore crucial (24). However, research on school-to-work transitions among vulnerable or at-risk youth, including young people with special educational needs, remains limited, particularly with regard to their longer-term career trajectories (14).

Students in special needs education may be particularly vulnerable during the school-to-work transition, as they often face distinct challenges and additional barriers compared to their peers in mainstream education. Moreover, they may be more likely to experience further obstacles linked to their intersecting social identities. Evidence from large-scale register data in The Netherlands shows that, after mainstream secondary vocational education, women and graduates with a migration background are less likely to enter and maintain paid employment (25). Whether similar, or perhaps even amplified, sociodemographic disparities exist among former special needs education students remains unclear. These findings underscore the importance of examining employment participation both across and within educational tracks in order to explore how gender and migration background relate to different patterns of post-school employment trajectories.

Understanding how educational pathways shape labor market access is essential for identifying where and how to intervene. Yet, within this already disadvantaged group, it remains unclear who are most vulnerable to exclusion. This study addresses that gap by examining the employment participation of individuals transitioning from secondary special needs education, with the aim of uncovering patterns of vulnerability to inform strategies to promote inclusive employment policies. Specifically, the study aims to: (i) assess employment participation among individuals transitioning from a special needs type education to the labor market, (ii) identify and describe latent employment participation trajectories within this population over time, and (iii) examine the extent to which gender and migration background are associated with these trajectories.

## Methods

### Data and study population

This longitudinal study, conducted using registry data from Statistics Netherlands, examined the transition from secondary special needs education and secondary practice-based education to the labor market, with a maximum follow-up of seven years and an average follow-up of four years. Informed consent was not required for this study as Dutch legislation permits affiliated research institutes to use the pseudonymized registry-based data. The Medical Ethical Committee of Erasmus MC Rotterdam approved the current study (MEC-2023-0708).

Individuals who were enrolled in either secondary special needs education or secondary practice-based education at any point between 2016 and 2023 were identified in the registry. Subsequently, individuals who transitioned out of either educational pathway during the follow-up period were selected (N=129 534). Individuals were excluded if they were younger than the minimum age required to be considered part of the labor force at the moment of transition (≥15 years) (N=4448) or if the distinct track within special needs education could not be determined (N=108). Subsequently, 74 669 individuals were selected following a transition out of secondary special needs education, and 50 309 individuals following a transition out of secondary practice-based education (supplementary material, URL, figure S1). Together, secondary special needs education and secondary practice-based education currently account for approximately 7.4% of all secondary education students in The Netherlands (4.2% and 3.2%, respectively) (26).

### Education

The last school year in which an individual was enrolled in either type of secondary education was determined from the registry data. The transition out of secondary education (baseline) was defined as occurring on the final day of that school year as the exact date of transition could not be determined. The type of special needs education was determined based on the available information for the final year of enrollment.

The Dutch secondary education system is characterized by relatively early tracking and institutional differentiation between educational pathways. Similar early tracking structures are observed in several continental European education systems, including Germany and Belgium, where students may be allocated to distinct lower secondary tracks at a comparatively young age (27).

### Secondary special needs education

In The Netherlands, secondary special needs education offers three distinct tracks, each defined by the anticipated transition pathway a student is likely to take upon exiting. Students who, despite their disabilities or limitations, are expected to obtain a diploma in regular secondary education with additional support, follow the ‘post-secondary education track’. This track is designed to prepare students for continued studies in tertiary education. Students allocated to the ‘labor market integration track’ are prepared for direct entry into paid employment as they are unlikely to obtain a diploma in regular education due to their disabilities or limitations. The track focuses on developing professional skills by offering practical support and facilitating internships. Lastly, the ‘sheltered day programs track’ is intended for students who are not expected to generate independent income, even within a supported work environment. This track primarily focuses on students with severe and multiple disabilities and aims to enhance self-reliance.

### Secondary practice-based education

Alternatively, students who do not meet the cognitive demands of mainstream secondary education, but do not require the intensive support offered in secondary special needs education, are referred to regular ‘practice-based’ secondary education. This educational track emphasizes practical, hands-on learning, with a strong focus on the development of general vocational skills in order to prepare students for direct entry to the labor market. Admission to ‘practice-based’ secondary education is determined based on IQ score of 50–80 and the extent of the student’s learning delay (≥3 years). As such, there is considerable overlap in both educational content and target population between this form of education and the labor market-oriented pathway within secondary special education (28).

### Employment status

Employment status was defined monthly, utilizing the indicator for main source of income in Statistics Netherlands. Individuals whose highest source of income in the reference month was paid employment, self-employment, or work in a family business without a formal employment agreement were classified as employed. As this measure is based on the primary income source rather than working hours, it does not distinguish between part- and full-time employment. For descriptive purposes, employment status was categorized in three groups: employed, in education, or NEET (not in education, employment, or training). For the group-based trajectory modeling, employment status was subsequently dichotomized as employed versus not employed.

### Sociodemographic characteristics

Register data concerning age, gender and migration background were obtained at baseline. The register provides dichotomic gender data (man/woman) as recorded in the Personal Records Database. In the event of a gender change, only the most recently registered gender is retained in the database. Individuals born outside The Netherlands or with at least one parent born abroad were identified as having a migration background. To adopt an intersectional analytical perspective, gender and migration background were operationalized as a combined four-category variable: men without migration background, men with migration background, women without migration background, and women with migration background.

### Statistical methods

For each of the included educational tracks, monthly prevalence of three key labor market outcomes: employment, continued education, or NEET was computed. Stacked density plots were used to visualize labor market participation dynamics within each group and to facilitate comparisons between groups.

Group based trajectory modelling (GBTM) was hereafter used to identify clusters of individuals with similar work participation trajectories following their transition from education to the labor market. Latent trajectories were identified separately for each educational track and used for profiling the sociodemographic characteristics (gender, migration background) of individuals within each cluster. The process of GBTM was conducted following the *Group Based Trajectory Modelling: Methodological Guide*, commissioned by the European Union Erasmus+ program (29).

Models with progressively increasing group counts were tested, and model fit was evaluated using the Bayesian Information Criterion. Regardless of the Bayes statistics, models were excluded from consideration if any trajectory group represented <5% of the total cases, or if the model failed to capture new distinctive features of the data. Polynomial functions were subsequently tested to determine the optimal shape for each trajectory, starting with a cubic form and progressively simplifying to quadratic or linear if the higher-order polynomial term proved non-significant (30, 31). Model adequacy was further evaluated using the average posterior probability of assignments (APPA), with a threshold of ≥0.7, and the odds of correct classification (OCC), with a minimum of 5.0 for each trajectory group (32). The fit statistics for the selected models along with considered alternatives models are presented in supplementary table S1.

Individuals were accordingly assigned to a trajectory group based on their highest posterior probability of group membership. Descriptive statistics were presented to illustrate the sociodemographic composition of the identified latent groups. Multinomial logistic regression analyses were thereafter conducted to examine the associations between the combined gender-migration variable and trajectory group membership. To formally assess statistical interaction, additional models including a gender × migration background interaction term were estimated and evaluated using a joint Wald test. All regression models were adjusted for age. The analyses were conducted in STATA version 16.1, utilizing the TRAJ package (33).

## Results

A total of 74 669 individuals were identified as transitioning out of secondary special needs education between 2016 and 2023, following one of the three distinct educational tracks. The majority transitioned through the post-secondary education track (N=39 448) followed by the labor market integration (N=20 275) and the sheltered day programs (N=14 946) tracks. Additionally, 50 309 individuals were identified as transitioning out of regular secondary education through the practice-based education track during the same period.

The mean age at transition varied by educational track (range 17.1–18.3 years, table 1). Across all groups, the majority of students were men, with the highest proportion observed in the labor market integration track (73.4%) and the smallest in the practice-based education track (58.3%). The proportion of individuals with a migration background was highest in the practice-based education track (43.2%) and lowest in the post-secondary education track (24.8%).

**Table 1:** Baseline characteristics of the study population, stratified by type of secondary education and educational tracks.[SD=standard deviation.]

	Secondary special needs education									Secondary regular education	
	Labor market integration track (N=20 275)			Post-secondary education track (N=39 448)			Sheltered day programs track (N=14 946)			Practice-based track (N=50 309)	
	Mean (SD)	N (%)		Mean (SD)	N (%)		Mean (SD)	N (%)		Mean (SD)	N (%)
Age in years	17.8 (1.3)			17.1 (1.2)			18.3 (1.1)			17.6 (0.9)	
Female gender		5397 (26.6)			11 281 (28.6)			5693 (38.1)			20 997 (41.7)
Migration background		6503 (32.1)			9772 (24.8)			4252 (28.4)			21 754 (43.2)

**Figure 1:**
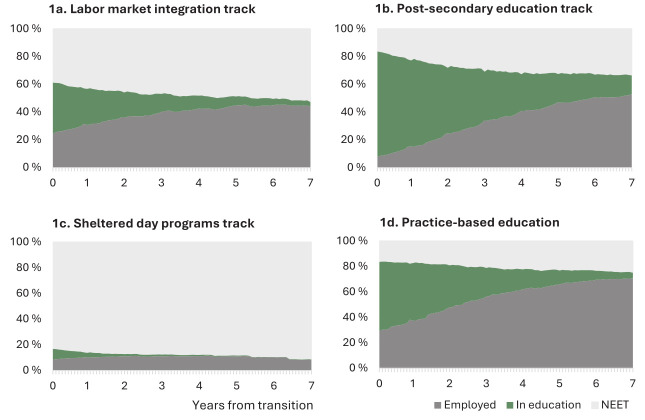
Dynamic changes in labor market participation following a transition out of secondary (special needs) education: a seven-year follow-up.

Figure 1 shows that among individuals who transitioned through the labor market integration track (1a), the proportion in paid employment steadily increased during the first three years, reaching 40%, and subsequently stabilized at 44–45%. In contrast, individuals from the post-secondary education track (1b) initially had a lower proportion in paid employment than the labor market integration group, but this proportion exceeded that of the labor market group after 4.5 years and continued to rise, reaching 53% by the end of follow-up. The highest proportion in paid employment was continuously observed in the practice-based education group (1d), peaking just over 70% in the final year of follow-up, whereas the lowest proportion in paid employment was found in the sheltered day programs track (1c), at approximately 10% throughout the follow-up period.

The share of individuals classified as NEET gradually increased within the labor market integration group, rising from an initial 39% to 52% by the end of follow-up. Among individuals who transitioned via the sheltered day program track to the labor market, 91% were classified as NEET after seven years. In contrast, individuals from the post-secondary education track and practice-based education were less likely to be NEET at the end of follow-up, with rates of 33% and 25% respectively.

**Figure 2:**
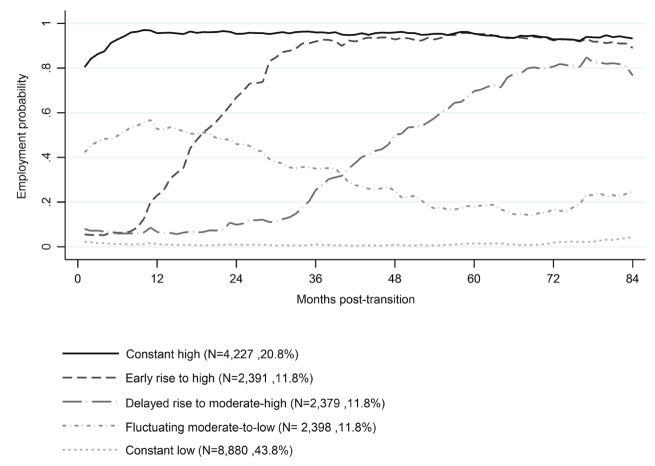
Group-based trajectories of employment probability following a transition out of secondary special needs education – the labor market integration track - (N=20 275).

Among individuals from the labor market integration track, five latent trajectory groups were identified and labelled based on observed patterns of employment participation over time (figure 2). The largest group (43.8%) exhibited consistently low employment participation throughout the follow-up period. In contrast, a second group (20.8%) maintained high levels of paid employment from the onset and throughout, while others (11.8%) gradually increased in employment participation, reaching comparably high levels after approximately four years. Another group of equal size (11.8%) demonstrated persistently fluctuating low-to-moderate levels of employment participation across the follow-up period. Supplementary figures S2, S3, and S4 present the latent trajectory analyses across all other educational groups.

Men were more strongly represented in the relatively favorable groups, particularly ‘constantly high’ (81.4%) and ‘early rise’ (81.2%), while women were more prevalent in the ‘constantly low’ group (33.3%). The ‘constantly high’ group also had the highest proportion of individuals aged ≥18 at the time of transition (75.1%), whereas the younger age group (15–17 years) was most represented in the ‘delayed rise’ and ‘early rise’ groups. Having a migrant background was more common in the less favorable trajectories, especially among those in the ‘delayed rise’ (39.6%), ‘fluctuating’ (35.6%), and ‘constantly low’ (34.5%) groups (table 2). Additional information on the composition of the identified latent trajectory groups in all other educational tracks is provided in supplementary tables S2, S3, and S4.

**Table 2:** Sociodemographic composition of the secondary special needs education ‘labor-market-integration track’, and its’ (five) identified latent trajectory groups based on employment participation following a transition to the labor market.

		Total (N=20 275)		Employment participation trajectory groups								
				Constant high (N=4227)		Early rise to high (N=2391)		Delayed rise to moderate-high (N=2379)		Fluctuating moderate-to-low (N=2398)		Constant low (N=8880)
		%		%		%		%		%		%
Baseline age												
	15–17	39.9		23.4		54.2		53.9		39.4		40.2
	≥18	61.1		76.6		45.8		46.1		60.6		59.8
Gender												
	Men	73.4		81.4		81.2		72.5		77.2		66.7
	Women	26.6		18.6		18.8		27.5		22.8		33.3
Migrant background												
	No	67.9		78.0		70.2		60.4		64.4		65.5
	Yes	32.1		22.0		29.8		39.6		35.6		34.5

Table 3 shows that, compared to men with no migration background, all other subgroups had a higher relative risk of belonging to less favorable work participation trajectories. For example, women without migration background were more likely to belong to the most unfavorable trajectory group, characterized by consistently low employment participation [relative risk rate (RRR) 2.57, 95% confidence interval (CI) 2.31–2.85]. In addition, men with a migration background were more likely to experience a delayed trajectory towards moderate-high employment participation (RRR 2.85, 95% CI 2.51–3.24) and fluctuating employment participation (RRR 2.30, 95% CI 2.03–2.62). However, the combined effect of gender and migrant background was smaller than would be expected based on the multiple of their individual effects. Nevertheless, women with a migration background had the highest probability of being in the group with constantly low employment participation (RRR 3.26, 95% CI 2.79–3.81).

**Table 3:** Predictors of latent trajectory group membership following a transition from the secondary special needs education ‘labor-market-integration track’: the role of gender and migration background. [RRR=risk rate rate; CI=confidence interval.]

Gender and migration background	Employment participation trajectory groups											
	Constant high (N=4227)	Early rise to high (N=2391)			Delayed rise to moderate-high (N=2379)			Fluctuating moderate-to-low (N=2398)			Constant low (N=8880)	
		RRR	95% CI		RRR	95% CI		RRR	95% CI		RRR	95% CI
Men/no migration background	Reference	1.0	-		1.0	-		1.0	-		1.0	-
Women/no migration background	Reference	1.12	0.96–1.30		2.06	1.78–2.38		1.51	1.30–1.75		2.57	2.31–2.85
Men/migration background	Reference	1.70	1.50–1.94		2.85	2.51–3.24		2.30	2.03–2.62		2.24	2.03–2.47
Women/migration background	Reference	1.41 ^a^	1.13–1.76		3.12 ^a^	2.55–3.80		2.00 ^a^	1.62–2.46		3.26 ^a^	2.79–3.81

^a^ Interaction between sex and migration background is statistically significant at the 0.05 level. Adjusted for age.

Similar patterns were observed across all other education groups (supplementary table S5, S6 and S7). Both gender and migration background were independently associated with an increased relative risk of belonging to less favorable employment participation trajectories. Specifically, being a woman or having a migration background significantly increased the likelihood of having consistently low employment participation, experience fluctuating employment patterns, or face delayed entry into moderate-to-high employment compared to men with no migration background across all educational tracks. The most pronounced gender disparities were found among former practice-based education students, where women were disproportionately more likely to experience consistently low employment participation. These disparities were even greater among women with a migration background (RRR=13.21, 95% CI 12.15–14.26).

## Discussion

Overall, the findings reveal a generally low level of employment participation among former special needs students, with employment participation not exceeding 53% following secondary special needs education tracks during the first seven years after leaving school. However, there is substantial heterogeneity in employment participation following the distinct educational tracks that provide additional support to students with special educational needs. Employment was initially highest among those transitioning from the ‘labor market integration’ and ‘practice-based education’ track. However, while employment participation for the labor market integration group plateaued at around 45%, it continued to rise among former ‘post-secondary’ and ‘practice-based’ education graduates, ultimately resulting in substantially higher employment participation. Within each educational track, distinct latent employment trajectories were identified, underscoring the diversity of post-school pathways within a single track and revealing early risk profiles for labor market integration. Gender and migration background were strongly associated with these latent trajectories: women and individuals with a migration background faced markedly higher risks of following less favorable employment patterns. These findings highlight that educational pathways alone do not fully account for differences in labor market outcomes. Instead, sociodemographic factors intersect with educational pathways to shape long-term labor market integration.

This study highlights the long-term NEET risks linked to a transition from the different secondary education tracks in special needs settings and regular practice-based education. Among those who entered the labor market via the ‘labor market integration’ track, 52% were classified as NEET seven years later. In contrast, students from the ‘post-secondary’ and ‘practice-based education’ tracks had significantly lower NEET rates of 33% and 25%, respectively. The gap between the labor market integration and practice-based group is striking, given their shared vocational focus (direct labor market entry) and similar target populations. These high NEET rates are particularly concerning as sustained NEET status during the early career phase may represent not only an economic vulnerability but also a potential precursor to longer-term health and social disadvantage (21, 34). Our findings align with previous findings within the Dutch context, showing that practice-based students show better long-term outcomes than students in secondary special needs education, including higher rates of continued education, employment, and attainment of start qualifications (28). It has been suggested that the elevated NEET risk among former special needs students cannot be fully explained by differences in socio-demographic background or cognitive and non-cognitive skills. About half of the risk appears to stem from the stigma of the special needs label, which may reduce how employers and training providers perceive qualifications (6). While these findings clearly show that track choice matters, a recent Dutch report highlights that placement decisions between special needs and regular practice-based education are often influenced by practicalities such as school proximity rather than pedagogical fit, especially for students whose support needs could be met in either setting (28). These findings imply that educational track assignment may not always reflect students’ best long-term interests, and more deliberate guidance is needed to avoid reinforcing disadvantage.

In our cohort, women with former special education needs were consistently more likely than men to follow less favorable employment trajectories and experience prolonged periods of unemployment in the initial years following their transition to the labor market. This finding aligns with previous research showing that young women with disabilities generally face lower employment rates, earn lower wages, and have fewer opportunities for career advancement than their male peers, despite attaining comparable educational qualifications (35, 36). These gendered disparities in employment participation may have broader implications for health and well-being as limited access to paid employment has been shown to undermine mental health, delay independence, and further reduce opportunities for social participation among young women with disabilities (35, 37, 38). While previous research has highlighted the concept of “double jeopardy”, where women with disabilities face both gender stereotypes and disability-related discrimination (36), our findings suggest that migration background may constitute an additional layer of disadvantage, further limiting access to stable, high levels of employment participation. The persistence of these disparities suggests that existing vocational support structures may not equally reach or benefit all groups, particularly at the intersection of gender and migration background. Our findings therefore reinforce earlier calls for gender-sensitive vocational approaches that acknowledge and address the intersecting barriers young women face as they transition from secondary special needs education to the labor market (36, 39).

One of the strengths of this study is the use of national registry data, which ensures inclusion of the entire Dutch population that met the criteria of having attended secondary education with special needs provision between 2016–2023. A second strength is that the study does not treat all special needs students as a single group but instead distinguishes between the various educational tracks within special needs education. This approach provides a more nuanced understanding of outcomes across different pathways. While working with registry data typically provides a broad overview, it also comes with inherent limitations in depth and detail. In particular, information on the nature or severity of individuals’ disabilities is not available in the registry data from Statistics Netherlands. The type of educational track followed may serve as a rough proxy for support needs, and likely reflects underlying differences in health conditions and functional limitations. Consequently, observed differences in employment trajectories between tracks may partly reflect selection processes related to underlying health status and functional capacity, rather than the impact of track orientation alone. These limitations underscore the need for complementary research linking educational data with more detailed health information.

A key strength of the applied method (group-based trajectory modeling) is its’ ability to identify subgroups of individuals who follow similar patterns over time. However a limitation concerns the inherent subjectivity involved in selecting the optimal number of trajectory groups. While statistical-fit criteria can guide model selection, final decisions still rely on qualitative judgment. In particular, researchers must assess whether the inclusion of an additional trajectory group reveals meaningfully distinct and interpretable features of the data, especially because the statistical fit commonly continues to improve with each added group (32). Ultimately, the objective of group-based trajectory modeling is not merely to maximize statistical fit but to identify distinct and meaningful patterns in the underlying distribution. As noted by Nagin and colleagues (40), “a final chosen model should include the fewest groups necessary to capture distinctive trajectory clusters that are relevant to the research question of interest.” Nevertheless, different researchers may arrive at different conclusions regarding the optimal number of groups, which may complicate the reproducibility of studies using group-based trajectory modeling. To address this limitation, we have included the fit statistics for both the selected models and all considered alternatives in the supplementary materials (supplementary table S1).

Moreover, in this study, the associations between gender, migration background, and latent trajectory group membership were examined using post-hoc multinomial regression models, rather than incorporating these variables directly into the group-based trajectory model. Consequently, gender and migration background did not influence the estimation of the latent trajectories themselves, allowing time-varying employment status to remain the sole determinant of trajectory classification. This two-step approach was chosen to preserve interpretability and comparability across educational tracks. However, it assumes that trajectory classification is final and does not formally account for uncertainty in group assignment. Nonetheless, model adequacy metrics, including APPA and OCC, indicated sufficiently high classification quality to justify this approach.

### Concluding remarks

This study revealed marked heterogeneity in employment outcomes across the distinct education tracks for students with special needs in The Netherlands, demonstrating that employment patterns differ substantially across early educational pathways. Long-term employment participation was notably higher for graduates from the secondary special needs track oriented toward continued education (post-secondary education track) and from secondary practice-based education. Strikingly, over half of the special needs students who attended the labor-market integration track were classified as NEET seven years post-transition. Given the well-established association between prolonged unemployment and adverse mental and physical health outcomes, such early labor market exclusion may have consequences that extend beyond economic participation, potentially contributing to cumulative socioeconomic and health disadvantage.

By identifying distinct early employment trajectories following secondary special needs education, this study highlights critical transition points where targeted support may reduce long-term labor market exclusion and associated health inequalities. Specifically, our findings point to the need for more intentional, equity-focused guidance in track assignment. Educational choices must prioritize long-term inclusion, not just short-term practicality. Moreover, they underscore the importance of coordinated support across educational and labor market context during the school-to-work transition.

Group-based trajectory modeling moreover revealed diverse post-secondary school employment patterns within each educational track. These findings demonstrate that educational pathways do not result in uniform labor market outcomes but instead reveal distinct early risk profiles for longer-term employment participation. Women and individuals with a migration background were consistently more likely to experience less favorable employment trajectories after transitioning from a secondary special needs education to the labor market. The persistence of these disparities suggests that existing transition support structures may insufficiently address intersecting vulnerabilities related to gender and migration background. Hence, these findings reinforce calls for gender-sensitive and culturally responsive vocational support services, tailored to the individuals’ identity and needs.

## Supplementary material

Supplementary material
