# Supplementary material for: From secondary special needs education to the labor market: latent trajectories and inequalities in employment participation
Source: Scand J Work Environ Health. 2026 Jun 26;52(4):381–90. doi: 10.5271/sjweh.4292 (PMC13344180; doi:10.5271/sjweh.4292)
Supplement: Supplementary material [file SJWEH-52-381-S001.pdf]

From secondary special needs education to the labor market: latent trajectories and inequalities in employment participation[1]

by Robert Ciliacus, MSc, Fabio Porru, PhD, Alex Burdorf, PhD, Merel Schuring, PhD [2]

1. Supplementary tables and figures
2. Correspondence to: Merel Schuring, Department of Public Health, Erasmus University Medical Center, PO box 2040, 3000 CA Rotterdam, The Netherlands. [E-mail: m.schuring@erasmusmc.nl]

Supplementary table 1. Fit statistics of group-based trajectory models by education track

Number of trajectory Groups	Polynomial functions	BIC	Smallest group (%)	Lowest APPA	Lowest OCC	New distinctive features
**Secondary special needs education: labor market integration track**						
2	3-3	-372885.03	40.99	0.99	55.95	-
3	3-3-3	-319131.85	25.65	0.94	18.74	Yes
4	3-3-3-3	-292172.71	17.50	0.91	14.78	Yes
5	3-3-3-3-3	-274724.25	12.08	0.88	11.36	Yes
**5**	**3-2-3-3-3**	-274719.36	**12.08**	**0.88**	**11.38**	**Yes**
6	3-3-3-3-3-3	-265094.38	9.01	0.86	10.82	No
**Secondary special needs education: Post-secondary education track**						
2	3-3	-683746.20	38.57	0.95	11.96	-
3	3-3-3	-607208.19	21.03	0.81	4.56	Yes
4	3-3-3-3	-566983.50	12.54	0.79	5.28	Yes
**4**	**2-3-3-3**	**-566987.79**	**12.57**	**0.79**	**5.30**	**Yes**
5	3-3-3-3-3	-563770.72	6.85	0.76	7.5	No
**Secondary special needs education: Sheltered day programs track**						
2	3-3	-89758.51	13.36	0.97	6.21	-
3	3-3-3	-50525.68	5.80	0.99	53.80	yes
**3**	**2-2-3**	**-50519.52**	**5.80**	**0.99**	**51.84**	**yes**
4	3-3-3-3	-63686.66	6.60	0.42	1.05	no
**Regular secondary education: practice-based education track**						
2	3-3	-965468.75	44.86	0.98	47.87	-
3	3-3-3	-821165.18	31.30	0.85	12.88	yes
4	3-3-3-3	-756156.68	12.11	0.84	10.38	yes
**5**	**3-3-3-3-3**	**-703459.34**	**11.40**	**0.70**	**7.05**	**yes**
6	3-3-3-3-3-3	-682350.23	8.74	0.69	7.56	no

Bold= preferred model based on statistical fit and its' ability to identify new distinctive features of the data

Polynomial order: 1=linear, 2=quadratic, 3=cubic
BIC= Bayesian Information Criterion
Lowest APPA= Lowest mean posterior probability of group membership across all trajectory groups
Lowest OCC= Smallest odds ratio of correct classification across all trajectory groups

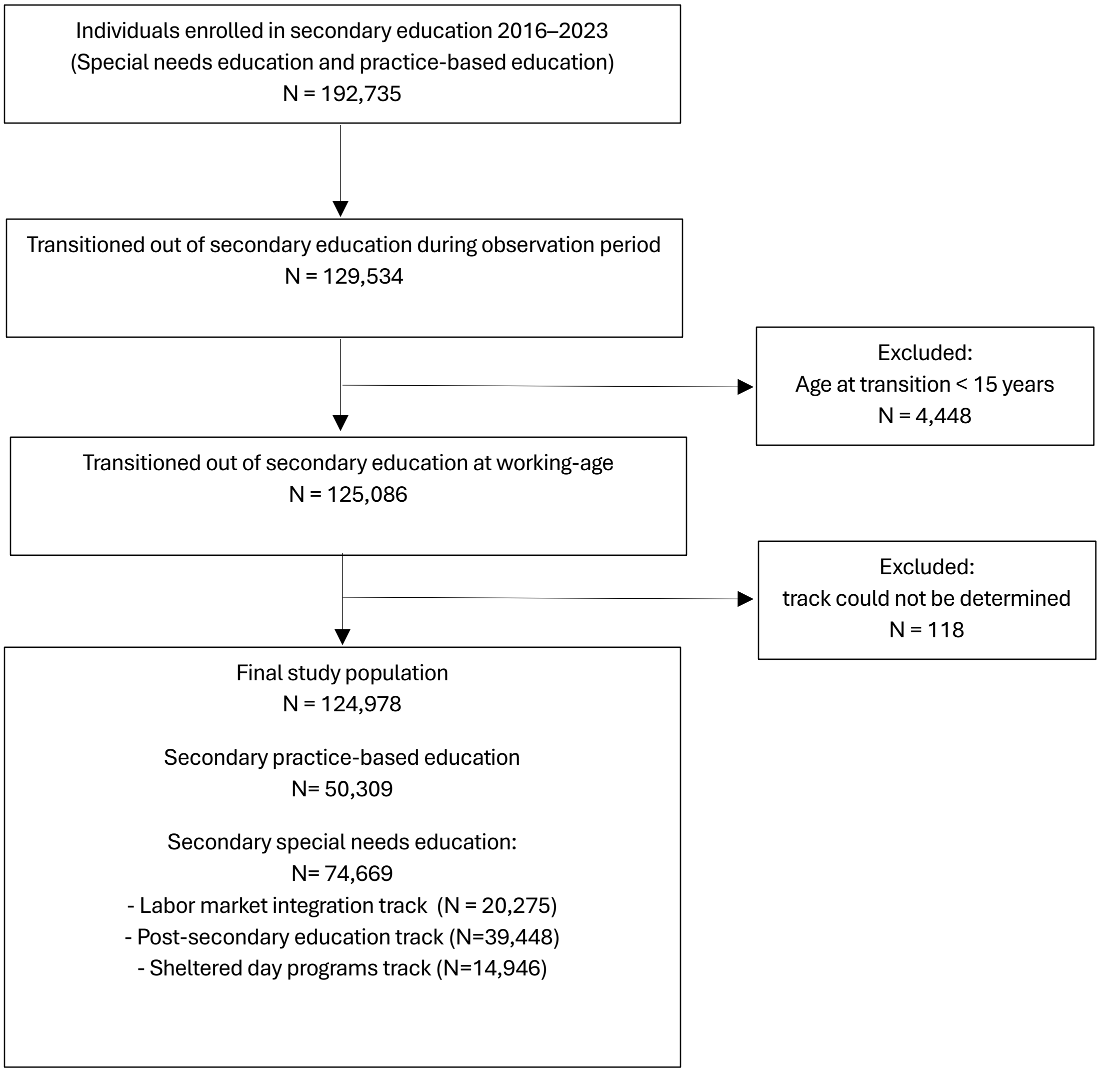

Supplementary Figure 1. Flowchart of study population selection from secondary special needs and practice-based education

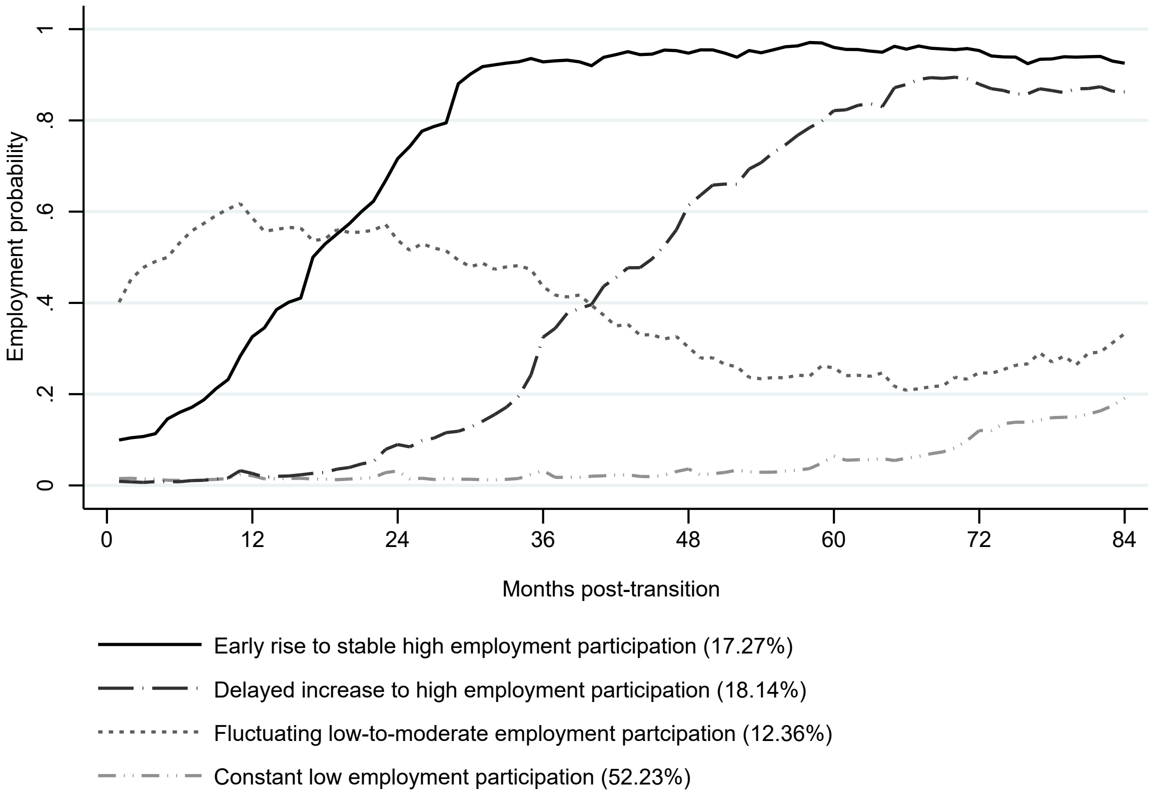

Supplementary Figure 2. Group-based trajectories of employment probability following a transition out of secondary special needs education 'post-secondary education' track (N=39,448).

Supplementary Table 2. Sociodemographic composition of the 'post-secondary education' track, and its' (four) identified latent trajectory groups based on employment participation following a transition to the labor market.

	Total (N=39,448)	Employment participation trajectory groups			
		Early rise to stable high (N= 6,811)	Delayed stable high (N= 7,156)	Fluctuating (N= 4,877)	Constant low (N= 20,604)
	%	%	%	%	
**Gender**					
Men	71.40	79.68	73.70	70.82	68.01
Women	28.60	20.32	26.30	29.18	31.99
**Baseline age**					
15 - 17	65.99	68.73	75.14	49.76	65.74
18 +	34.01	31.27	24.86	50.24	34.26
**Migrant background**					
No	75.23	80.41	76.62	73.51	73.44
Yes	24.77	19.59	23.38	26.49	26.56

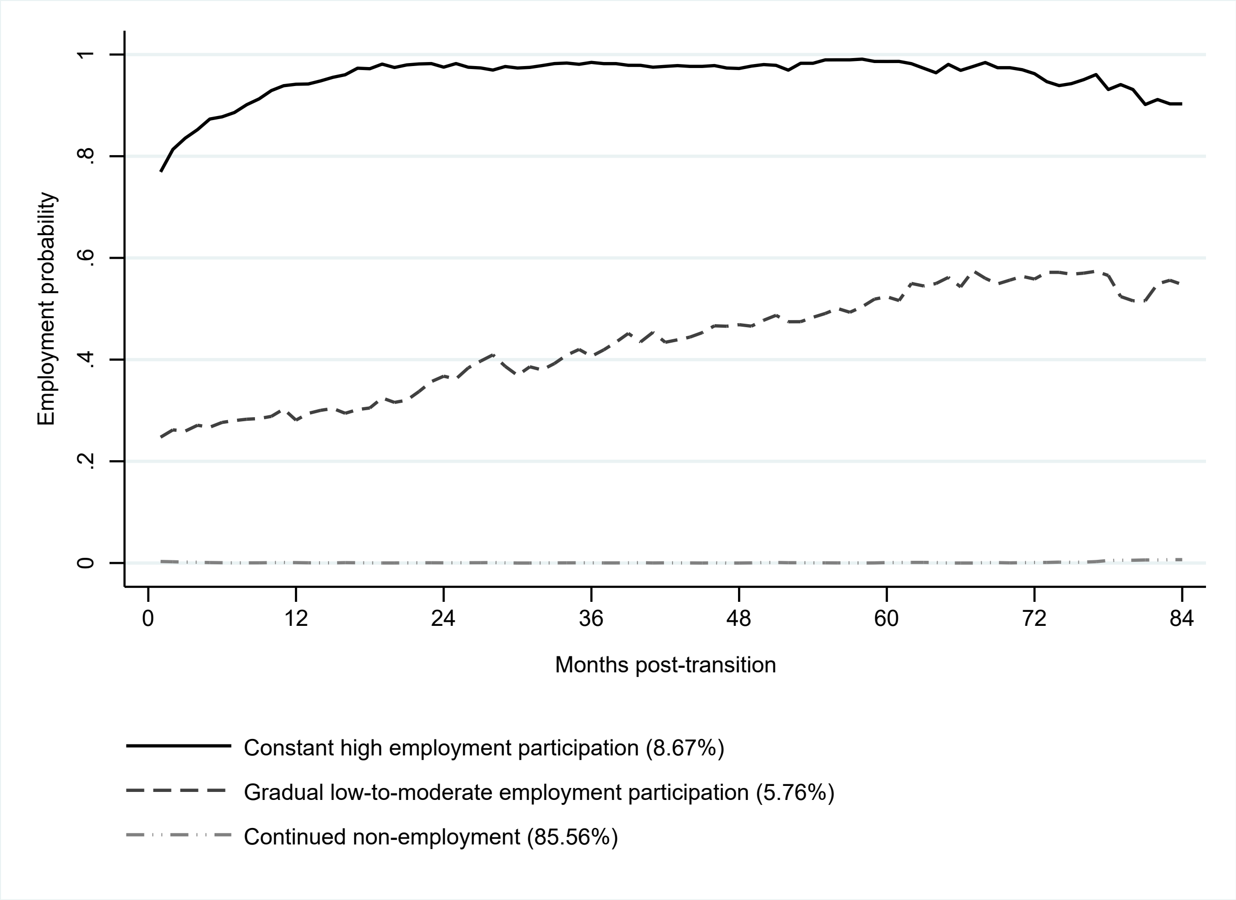

Supplementary Figure 3. Group-based trajectories of employment probability following a transition out of secondary special needs education 'sheltered day programs track' (N=14.946).

Supplementary Table 3. Sociodemographic composition of the secondary special needs education 'sheltered day programs track', and its' (three) identified latent trajectory groups based on employment participation following a transition to the labor market.

	Total	Employment participation trajectory groups		
		Early and Stable	Continued non-employment	Gradual entry
	(N=14.946)	(N=1.299)	(N=12.825)	(N=822)
	%	%	%	%
**Gender**				
Men	61,91	75,52	59,71	74,70
Women	38,09	24,48	40,29	25,30
**Baseline age**				
15 - 17	16,71	11,55	16,37	30,05
18 +	83,29	88,45	83,63	69,95
**Migrant background**				
No	71,55	72,13	72,16	61,19
Yes	28,45	27,87	27,84	38,81

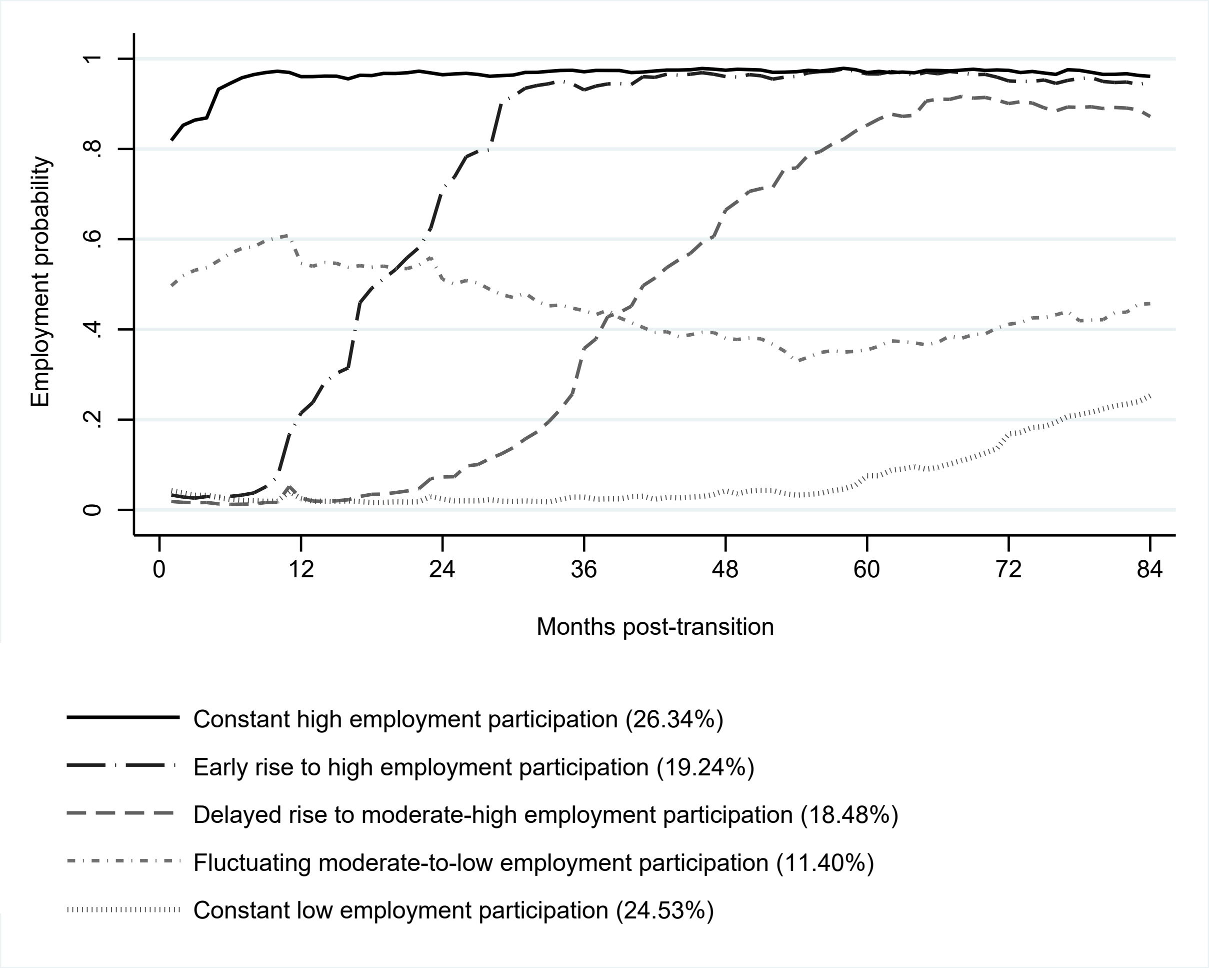

Supplementary Figure 4. Group-based trajectories of employment probability following a transition out of regular secondary education 'the practice-based education track' (N=50.309).

Supplementary Table 4. Sociodemographic composition of the secondary education 'practice-based education track', and its' (five) identified latent trajectory groups based on employment participation following a transition to the labor market.

	Total	Employment participation trajectory groups				
		Constantly high	Constantly low	Fluctuating	Delayed rise	Early rise
	(N=50.309)	(N=13.575)	(N=17.338)	(N=5.510)	(N=5.908)	(N=7.978)
	%	%	%	%	%	%
**Gender**						
Men	58,26	72,99	46.95	61,13	48,65	62,94
Women	41,74	27,01	53.05	38,87	51,35	37,06
**Baseline age**						
15 - 17	39,66	23,26	48,80	29,18	51,90	45,90
18+	60,34	76,74	52,20	70,82	48,10	54,10
**Migrant background**						
No	56,76	76,24	44,18	52,27	45,67	62,27
Yes	43,24	23,76	55,82	47,73	54,33	37,73

Supplementary Table 5. Predictors of latent trajectory group membership following a transition from secondary special needs education 'post-secondary education track': the role of sex and migration background.

	Employment participation trajectory groups						
	Early rise to stable high (N= 6,811)	Delayed stable high (N=7,156)		Fluctuating (N= 4,877)		Constant low (N=20,604)	
		RRR	95% CI	RRR	95% CI	RRR	95% CI
Men – No migration background	Reference	1.0	-	1.0	-	1.0	-
Men – Migration background	Reference	1.31	1.20 – 1.44	1.46	1.32 – 1.62	1.61	1.49 – 1.74
Women – No migration background	Reference	1.50	1.37 – 1.64	1.58	1.43 – 1.75	1.99	1.85 – 2.15
Women – Migration background	Reference	1.65	1.42 – 1.92	2.03	1.73 – 2.37	2.33*	2.06 – 2.65

** Interaction between sex and migration background is statistically significant at the 0.05 level. Adjusted for age.*

Supplementary Table 6. Predictors of latent trajectory group membership following a transition from the secondary special needs education 'sheltered day programs track': the role of sex and migration background**.**

	Employment participation trajectory groups				
	Early and stable (N=1.299)	Continued non-employment (N=12.825)		Gradual entry (N=822)	
		RRR	95% CI	RRR	95% CI
Men – No migration background	Reference	1.0	-	1.0	-
Men – Migration background	Reference	1.02	0.88 – 1.19	1.63	1.32 – 2.03
Women – No migration background	Reference	2.11	1.81 – 2.46	1.05	0.82 – 1.35
Women – Migration background	Reference	2.18	1.73 – 2.76	2.01	1.45 – 2.80

** Interaction between sex and migration background is statistically significant at the 0.05 level. Adjusted for age.*

Supplementary Table 7. Predictors of latent trajectory group membership following a transition from the secondary regular education track 'practice-based education': the role of sex and migration background.

	Work participation trajectory groups								
	Constant high (N=13.575)	Constant low (N=17.338)		Fluctuating (N=5.510)		Delayed rise (N=5.908)		Early rise (N=7.978)	
		RRR	95% CI	RRR	95% CI	RRR	95% CI	RRR	95% CI
Men – No migration background	Reference	1.0	-	1.0	-	1.0	-	1.0	-
Men – Migration background	Reference	4.44	4.16 – 4.74	3.38	3.11 – 3.67	5.05	4.52 – 5.53	2.21	2.05 – 2.38
Women – No migration background	Reference	3.89	3.19 – 3.62	2.02	1.86 – 2.21	3.89	3.56 – 4.26	1.82	1.69 – 1.96
Women – Migration background	Reference	13.21*	12.15 – 14.36	4.88*	4.38 – 5.42	11.91*	10.72 – 13.24	3.07*	2.78 – 3.39

**Interaction between sex and migration background is statistically significant at the 0.05 level. Adjusted for age.*
